# Electrospun PCL Nerve Wrap Coated with Graphene Oxide Supports Axonal Growth in a Rat Sciatic Nerve Injury Model

**DOI:** 10.3390/pharmaceutics16101254

**Published:** 2024-09-27

**Authors:** Meaghan E. Harley-Troxell, Richard Steiner, Steven D. Newby, Austin J. Bow, Thomas J. Masi, Nicholas Millis, Alicia Adina Matavosian, Dustin Crouch, Stacy Stephenson, David E. Anderson, Madhu Dhar

**Affiliations:** 1 Laboratory of Tissue Engineering and Regenerative Medicine, Department of Large Animal Clinical Sciences, College of Veterinary Medicine, University of Tennessee, Knoxville, TN 37996, USA; mharley4@vols.utk.edu (M.E.H.-T.); rcsteiner925@gmail.com (R.S.); snewby@vols.utk.edu (S.D.N.); bow.austin@yahoo.com (A.J.B.); nmillis.dvm@gmail.com (N.M.); dander48@utk.edu (D.E.A.); 2 Department of Surgery, University of Tennessee Graduate School of Medicine, Knoxville, TN 37996, USA; tmasi@utk.edu; 3 Department of Mechanical, Aerospace, and Biomedical Engineering, University of Tennessee, Knoxville, TN 37996, USA; aam346@cornell.edu (A.A.M.); dcrouch3@utk.edu (D.C.); 4 Department of Plastic and Reconstructive Surgery, University of Tennessee Medical Center, Knoxville, TN 37920, USA; sstephenson@utmck.edu

**Keywords:** PCL, nerve wrap, graphene oxide, axonal support, rat sciatic nerve defect model, mesenchymal stem cells

## Abstract

**Background/Objectives:** Peripheral nerve injuries (PNIs) are a debilitating problem, resulting in diminished quality of life due to the continued presence of both chronic and acute pain. The current standard of practice for the repair of PNIs larger than 10 mm is the use of autologous nerve grafts. Autologous nerve grafts have limitations that often result in outcomes that are not sufficient to remove motor and sensory impairments. Bio-mimetic nanocomposite scaffolds combined with mesenchymal stem cells (MSCs) represent a promising approach for PNIs. In this study, we investigated the potential of an electrospun wrap of polycaprolactone (PCL) + graphene oxide (GO), with and without xenogeneic human adipose tissue-derived MSCs (hADMSCs) to use as a platform for neural tissue engineering. **Methods:** We evaluated, in vitro and in vivo, the potential of the nerve wrap in providing support for axonal growth. To establish the rat sciatic nerve defect model, a 10 mm long limiting defect was created in the rat sciatic nerve of 18 Lewis rats. Rats treated with the nanocomposites were compared with autograft-treated defects. Gait, histological, and muscle analyses were performed after sacrifice at 12 weeks post-surgery. **Results:** Our findings demonstrate that hADMSCs had the potential to transdifferentiate into neural lineage and that the nanocomposite successfully delivered hADMSCs to the injury site. Histologically, we show that the PCL + GO nanocomposite with hADMSCs is comparable to the autologous nerve graft, to support and guide axonal growth. **Conclusions:** The novel PCL + GO nerve wrap and hADMSCs used in this study provide a foundation on which to build upon and generate future strategies for PNI repair.

## 1. Introduction

Peripheral nerve injuries (PNIs) are a condition of the peripheral nervous system caused by trauma or disease, where approximately 3% of all trauma patients have a PNI [1,2]. In 2018, the National Institute of Neurological Disorders and Stroke (NINDS) estimated 20 million US citizens are currently affected by a PNI [1,3]. Neurotmesis, one of the most damaging forms of PNIs, affects 2.6% and 1.2% of upper and lower extremity trauma patients, respectively, around the globe and is often classified as grade 4 or 5 on the Sunderland nerve injury classification scheme [4,5]. Currently, the clinical standard of care for the repair of neurotmesis is the use of autograft nerve segments [5,6]. This method, however, is limited in the degree of healing. This strategy usually involves donor-site morbidity, sensory loss, scarring at the donor site, and neuroma formation [7,8]. The low quality of recovery for neurotmesis cases results in a large number of patients affected by chronic symptoms of peripheral neuropathy, contributing to an overall decrease in the wellbeing of the population. A corresponding increase in health care costs expended for the repair and management of these cases are currently estimated at around USD 150 billion annually [9]. PNI is a condition that leads to serious and long-term functional and physiological disabilities [5,10,11]. This presents a pressing need to research new alternative strategies for nerve repair for human medicine.

Tissue engineering is a growing field of science that has the potential for improving the rate and quality of healing damaged tissue such as that which occurs in PNIs. Peripheral neurons can spontaneously form axons in cases of mild damage [5]. However, novel therapeutic strategies of tissue engineering need to be implemented when it comes to large defects, which are unable to heal. Neural tissue engineering, which involves the combination of scaffolds with tissue-like and porous structures, can serve as a bridge between the two ends of the defect. Mesenchymal stem cells (MSCs), with a potential to differentiate into multiple cell lineages, may serve as an efficacious therapeutic modality to support axonal growth and thus promote nerve healing [12,13]. Hence, in nerve regeneration, it could be advantageous to combine these two components into a nerve wrap that can serve to support and guide axonal growth and elongation across the site of injury.

There are a variety of commercially available nerve wraps and tubular conduits intended to provide support and directional growth cues and prevent the loss of pro-regenerative trophic factors away from the repair site, thereby enhancing axonal growth and functional recovery. Examples include, NeuraWrap^™^ Nerve Protector (Integra LifeSciences, products.integralife.com), Axoguard Nerve Protector^®^ (Axogen, axogeninc.com), and NeuroMend (Stryker, stryker.com) [14]. These devices contain collagen as the bioactive factor, reducing the risk of cytotoxicity to the native tissue. Although these wraps minimize some complications, none directly promote nerve repair.

Graphene is a monolayer of sp2-hybridized carbon atoms first isolated from graphite [15]. Due to its particle size and dimensions, graphene demonstrates electrical and thermal conductivity, exceptional mechanical resistance, and flexibility [16]. Recent research has demonstrated that graphene-based nanocomposites have the potential to support peripheral nerve restoration [15,17,18,19,20,21]. These include manually generated or 3D printed graphene nanocomposite scaffolds, in which graphene can be combined with other polymeric substances such as polycaprolactone (PCL) or poly (lactic-co-glycolic acid) (PLGA). PCL fibers are commonly used as a base polymer in nerve tissue engineering as they are easily combined with other polymers and coatings for improved biocompatibility and mechanical properties, favored for promoting nerve repair and regeneration [22,23,24]. Similarly, bone marrow and adipose tissue-derived MSCs have been shown to differentiate into neural lineage while providing immunoregulatory effects to promote a microenvironment suitable for axonal growth [25,26]. Importantly, studies have shown that by using carbon-based nanomaterials, one can enhance the growth, survival, and differentiation of MSCs, supporting the notion that combining graphene-based nanoparticles with MSCs may enhance nerve repair [27,28]. Yet, with all the data published on research studies including graphene nanocomposites, clinical application has yet to be established. Based on the potential for graphene to play a role in nerve tissue engineering, this approach ultimately should yield commercial devices to improve the treatment of PNIs.

The purpose of this study is to investigate the potential use of a novel nerve wrap made of electrospun PCL coated with graphene oxide (GO) nanoparticles to improve nerve regeneration using a 10 mm long limiting-sized rat sciatic nerve defect model. We hypothesized that the presence of the oxidized form of graphene nanoparticles will create a microenvironment to support the neural differentiation of human adipose-derived MSCs (hADMSCs), which will subsequently support the in vivo axonal growth without any neuroma formation.

## 2. Materials and Methods

### 2.1. Materials

All biochemicals, cell culture supplements, and disposable tissue culture supplies were purchased from Thermo Fisher Scientific (Waltham, MA, USA), unless otherwise stated.

### 2.2. Graphene Oxide Dispersion and Fabrication of Electrospun Nerve Wrap

Single-layered GO with x-y dimensions of 300–800 nm and containing 35–45% oxygen content was commercially obtained (Cheap Tubes Inc., Grafton, VA, USA). The GO dispersion was generated as described by Waqar et al. [24]. Briefly, a 0.1 mg/mL single-layered GO dispersion was created by mixing 3.0 mg of GO with 30 mL of 18 MΩ deionized water. The dispersion was vacuum-filtered and washed with 18 MΩ deionized water several times to collect graphene precipitants. Dispersions were tip-sonicated for 2 h after filtration. Prior to use, the GO solution was bath-sonicated for 30 min to evenly disperse the nanoparticles that may have settled to the bottom of the container [29].

The nanocomposite of PCL and GO was prepared by electrospinning in a two-step process. A solution of 0.1 g/mL PCL (MW 80,000; Sigma-Aldrich, St. Louis, MO, USA) was prepared by dissolving 1.5 g of PCL powder in 15 mL 1:1 ratio of methylene chloride (MC) to dimethylformamide (DMF). The electrospinning apparatus was set up in a chemical fume hood with an Aladdin-6000 multi-barrel programmable 6 syringe pump (World Precision Instruments, Sarasota, FL, USA), a 5 mL syringe, a 19-gauge needle head, a voltage generator attached to the needle head, and a rotating mandrel collector wrapped with aluminum foil grounded to the fume hood box. PCL alone was electrospun at a 16–17.5 kV charge with the needle tip-to-collector distance set to 7 inches and the solution pumping rate set to 1 mL/hr. Each electrospinning process used 4 mL of the 0.1 g/mL PCL solution to create PCL meshes of random fiber orientation. In order to create PCL fiber meshes of greater rigidity and make them easier to manage when wetted in cell culture media, the solution was left to spin for 4 h, which used a total of 4 mL of spinning solution per mesh, which created a mesh of approximately 0.01 mm thickness. Next, 10 mL of 0.1 mg/mL of the GO solution was evenly distributed onto the surface of the 127 mm × 84 mm PCL mesh by airbrushing the GO solution using a Paasche airbrush (Paasche Airbrush H-Set Single Action Siphon Feed Airbrush kit, Kenosha, WI, USA) set to 21 psi on a hot plate at 45–55 °C. Meshes were further cut into 12 mm × 6 mm sections for in vivo applications. Each coated PCL mesh thus contained a concentration of 0.1 mg/mL of GO. The size was based on the expected sciatic nerve defect with a 1 mm margin at both proximal and distal ends for suturing. All sections were sterilized under UV light for 4 h prior to use [29].

### 2.3. Isolation and Ex Vivo Expansion of hADMSCs

Human adipose tissue was obtained from patients in accordance with an approved IRB protocol (# 3995) from the University of Tennessee, Knoxville. Informed client consent was obtained from the patient prior to the harvest. The isolation and characterization of the human primary adipose-derived mesenchymal stem cell line were established as described previously [30,31]. Cells were characterized and confirmed to be MSCs [30,31]. Cells were expanded and all experiments were performed in DMEM-F12 growth media (DMEM-F12 +10% fetal bovine serum +1% L-glutamine +1% penicillin streptomycin). Cells were kept in an incubator at 5% CO_2_ and 37 °C during the culture period. Regular media changes were performed every 2–3 days. All experiments were performed using cells from passage 2–6 in growth media. For all in vitro experiments, roughly 40,000 cells/well were seeded on the material or glass coverslips in a 24-well plate. One million cells were seeded onto each scaffold for in vivo analyses. Cells were left to adhere to the 2D and 3D substrates for at least 24 h before performing the in vitro assays or implantation into the nerve defect in vivo. All cells were DiI-labeled, as previously described, to confirm cell attachment to the nerve wrap prior to implantation in vivo [29,32].

### 2.4. Neural Differentiation and Expression of Neural Markers In Vitro

hADMSCs were evaluated for their potential to undergo neural differentiation using standard in vitro assays [33]. hADMSCs were DiI-labeled as previously described [32]. The red fluorescence allowed for the cells to be easily tracked on the dark graphene-coated PCL wrap. Roughly 40,000 DiI-labeled cells/well were seeded in a 24-well plate and differentiation was induced by supplementing the growth media with 0.5 mM of 3-isobutyl-1-methylxanthine (IBMX) and 1 mM of dibutyryl cyclic adenosine monophosphate (db-cAMP) (Sigma Aldrich, St. Louis, MO, USA). After 24 h, cells were fixed with 4% paraformaldehyde/PBS and were evaluated for the expression of specific neural markers, as reported previously [34]. The morphology and neural differentiation of hADMSCs were assessed using S100β expression (BD Biosciences, Franklin Lakes, NJ, USA). Subsequently, the expression of S100β was quantitated using Fiji/ImageJ 2.3.0/1.53q software [35,36]. All images were captured using a Keyence BZ-X710 All-in-one Fluorescent Microscope (Keyence, Itasca, IL, USA). Cells on scaffolds without the neural differentiation media (undifferentiated) and cells seeded on glass coverslips with the differentiation media served as controls [29].

### 2.5. Rat Sciatic Nerve Defect Model

All live animal experiments were performed as per approved protocol by the Institutional Animal Care and Use Committee (IACUC) at the University of Tennessee (IACUC Protocol #2574). Eighteen male 8-week-old Lewis rats were obtained commercially (Envigo, Indianapolis, IN, USA). Animals were randomly divided into 3 independent groups (n = 6 each) based on the treatments provided as follows: Group 1—PCL + GO wrap, Group 2—PCL + GO wrap + 1 million hADMSCs, and Group 3—autologous nerve graft. The autologous nerve graft was derived from the sciatic nerve segment that was excised from the treated limb during a single surgery. These segments were excised, reversed in directional alignment, and then sutured in place, serving as the autologous nerve graft. In all rats, a 10 mm long limiting-sized segmental defect was created by removing a segment of the sciatic nerve on the right hindlimb using methods previously described [37,38]. Briefly, under anesthesia, a 3 cm skin incision was made longitudinally to the posterior lateral aspect of each thigh, extending from the greater trochanter to the knee. A blunt dissection of the gluteus maximus and biceps femoris muscles exposed the sciatic nerve directly underneath. The 10 mm defect was created by sharp transection. Each rat was assigned to one treatment group according to pre-assignment as described above. The group of rats treated with the autologous graft served as the positive control, simulating clinical practice. The defect was enveloped in the nerve wrap, the proximal and distal nerve stumps were attached using 10-0 absorbable suture material (J&J MedTech, Diegem, Belgium), and a single stitch was used in the center to close the nerve wrap. Animals in each group were maintained for up to 12 weeks post-surgery with gait analysis data collection at 2-week intervals. At 12 weeks post-surgery, all rats were euthanized, and both hindlimbs were collected for nerve histological and muscle sarcomere assessments [29].

### 2.6. Gait Analysis 

Gait patterns were analyzed using a pressure sensor mat (Tekscan VH4, Tekscan, Boston, MA, USA) according to specifications previously reported [39]. Briefly, rats were trained to walk across the mat in a single direction for data collection. Pressure mat sensing was recorded once prior to surgery as baseline values, again on day 7 after surgery, and subsequently repeated at 2-week intervals through to the end of the 12-week study. Proprietary software, WALKWAY™ 7.7X, was used to analyze the data for stance time (seconds), stride length (cm), maximum force (kPa), and maximum force adjusted for body weight (%BW). *t*-tests were performed using GraphPad Prism software (version 10) to determine differences between the left (normal) and right (defect) hindlimbs (*p*-value ≤ 0.05) [29].

### 2.7. Histological Analysis

At 12 weeks, both hindlimbs were harvested. The left limb was used for muscle assessment as a contralateral comparison for the muscles of the treated right hindlimb. Affected nerve segments were collected from the right hindlimbs of all rats and aligned on sections of cardboard before being placed in 10% formalin to preserve the tissue sections’ alignment. Nerve defects included soft tissues proximal and distal to the gap and the nerve gap including either autologous grafts or nerve wraps. The tissues were embedded into paraffin and sectioned longitudinally for a histological analysis of the tissue segments extending from the proximal nerve stump to distal nerve stump. Histological assessment included routine hematoxylin and eosin (H&E) (Azer Scientific, Inc., Morgantown, PA, USA) to evaluate cellular detail, tissue architecture, and biocompatibility. All sections were evaluated by a board-certified pathologist. The MCOLL stain was used to assess the presence of myelin in the tissues and defects. MCOLL staining (Azer Scientific, Inc., Morgantown, PA, USA) involved the combined approach of using Luxol Fast Blue to stain for lipoproteins present on the surface of myelin sheath tissue and the Picrosirius Red stain for specific staining of different collagen tissues in the perineurium and epineurium sections of the nerves [40]. This combination allows for the clear identification of myelin tissue structures [29].

For quantitative assessment, each nerve section was imaged using a DMi1 Leica light microscope at 5× magnification (Leica Microsystems Inc., Deerfield, IL, USA). For each sample, multiple images were taken along the length of the nerve section with 20% overlap. The images were then stitched together to create a single image of the nerve. The stitched image was selected for the optimal contrast (green scale) and threshold (73). The nerve was isolated from the background and converted to a binary image of black (MCOLL-stained tissue) and white (unstained tissue). The ratio of black to white was calculated as the percent surface area, as an indicator of the area of positively stained MCOLL tissue as a percentage of the whole sectioned nerve. A one-way ANOVA test and Tukey’s multiple comparison test were performed using GraphPad Prism software (version 10) to determine statistically significant differences between the three treatment groups (*p*-value ≤ 0.05).

### 2.8. Muscle Analysis

The architecture of the triceps surae muscles, which are solely innervated by the sciatic nerve, was assessed. The triceps surae muscles comprise the lateral gastrocnemius, medial gastrocnemius, and soleus muscles. Hindlimbs were dislocated at the hip, skin was removed, and the entire limb fixed in 10% phosphate-buffered formalin for at least 5 days. All specimens were fixed in such a way that the knee and ankle joints were in approximately 90° flexion and neutral dorsi/plantar flexion (90° angle between the shank and foot), respectively. The triceps surae were dissected from the hindlimb, the distal tendon removed, and the three muscles separated from one another. The muscles were blotted dry for measuring the mass using a digital scale. Muscle length was measured using a digital caliper. An incision was made with a scalpel at the mid-belly and along the direction of pennation of the muscle fibers. Muscle fiber length and the pennation angle were measured at the incision site using a digital caliper and goniometer, respectively. Sarcomere lengths were measured from fibers excised from the muscle mid-belly using a standard laser diffraction technique [41]. From the muscle architecture measurements, the physiologic cross-sectional area (PCSA) and optimal fiber length were computed [42]. These values are proportional to the muscle force-generating capacity and active force-generating length range (i.e., excursion), respectively [43]. For calculating the PSCA, we assumed a skeletal muscle density of 1.054 g/cm [42,44]. We expressed all muscle architecture values as a percentage of values obtained on the contralateral non-operated side. For each muscle, percentages were compared among groups using a one-way ANOVA and post hoc Tukey HSD test with α = 0.05 [29].

## 3. Results

### 3.1. hADMSCs Adhered and Underwent Neural Transdifferentiation on Graphene Surfaces

**Figure 1:**
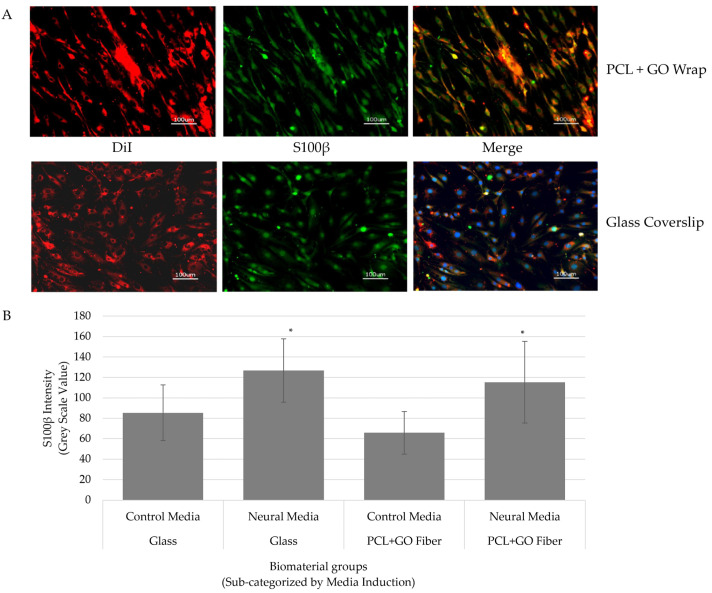
Neural differentiation of hADMSCs. Representative images showing the spatial and temporal expression of S100β when hADMSCs are seeded onto PCL + GO and the glass surfaces (**A**). Note the expression, as well as a discrete pattern of DiI-labeled hADMSCs, in the presence of cAMP containing differentiation media on graphene surfaces. Cells appear to be aligned on the PCL + GO surface compared to the random arrangement on the glass surface. These changes occur within 24 h post induction. S100β expression was demonstrated using immunofluorescence. S100β was further quantitated using Image J (**B**). Graph displays S100β intensity as measured on the different surfaces and under chemical induction. Significant differences were found between control media and neural media for both surfaces. * *p* ≤ 0.05.

Cell attachment and the neural differentiation of hADMSCs on PCL + GO surfaces were confirmed using immunofluorescence (Figure 1A,B). Cells were maintained in the standard DMEM F12 growth media, and when 70–80% confluent, were subjected to neural differentiation using a cAMP/IBMX cocktail for 24 h only. DiI-labeled hADMSCs adhered to the biomaterial surface and maintained their MSC morphology in the growth media. A routinely used glial protein marker, S100β, was expressed in both the control undifferentiated and differentiated cells seeded on both the glass coverslips, as well as graphene surfaces, confirming the cAMP/IBMX-mediated trans-differentiation of MSCs. This was further supported by the quantitative increase in the intensity of fluorescence between undifferentiated and differentiated cells (Figure 1B). There was no difference between the expression of S100β on the glass coverslips and the graphene substrates. Interestingly, there was a discrete difference in the spatial pattern of the expression of S100β between undifferentiated and differentiated cells and in the presence of various substrates. In the presence of the PCL + GO substrate, cells aligned in a specific pattern, which suggested not only neural differentiation but also a mechanoresponse of hADMSCs to the biomaterials within 24 h of exposure to the neural media.

### 3.2. Force Distribution and Surface Area Contact Ratios Were Maintained throughout the 12-Week Study

**Figure 2:**
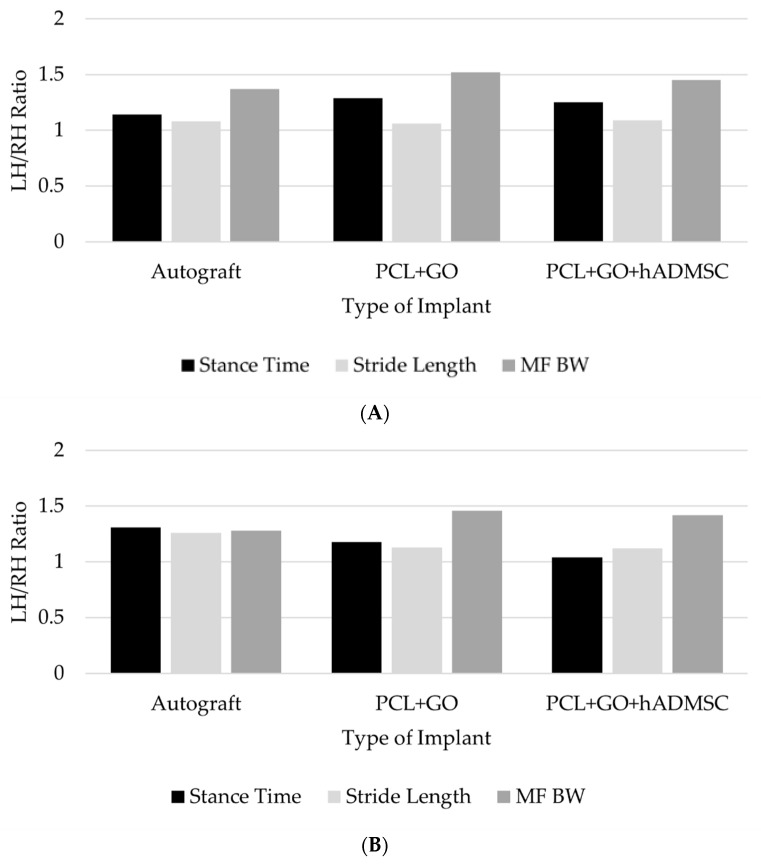
(**A**). Rat gait analysis at 2 weeks post-surgical nerve repair. Mean left hindlimb/right hindlimb (LH/RH) ratio for stance time, stride length, and maximum force (MF; adjusted for % body weight (BW)) by implant type. No statistically significant differences noted. (**B**). Rat gait analysis at 12 weeks post-surgical nerve repair. Mean left hindlimb/right hindlimb (LH/RH) ratio for stance time, stride length, and maximum force (MF; adjusted for % body weight (BW)) by implant type. No statistically significant differences noted.

Temporal data of rats did not show significant changes between the groups during the 12-week study period. Force distribution and surface area contact ratios, as determined by stance time, stride length, and maximum force between the right and left hindlimbs, were found to be similar to each other at all time points for all treatment groups. None of the treatment groups were significantly different from the baseline ratios of force distribution and surface area contact between the hindlimbs during the 12-week study period. This indicates that full functional repair did not occur in any nerve during the 12-week study period, and a longer time post treatment is potentially needed (Figure 2).

### 3.3. MCOLL-Stained Myelin Tissue in PCL + GO + hADMSC Treatment Group Is Comparable to the Autograft Group

The H&E evaluation indicated no local adverse reaction in all experimental groups. As expected, sciatic nerve tissue sections from the autograft group showed positive MCOLL staining with readily apparent tissue organization, as previously established [45,46,47,48,49,50]. The sections from the PCL + GO and PCL + GO + hADMSC experimental groups showed positive MCOLL staining, and tissue organization comparable with that of the autograft-treated group.

**Figure 3:**
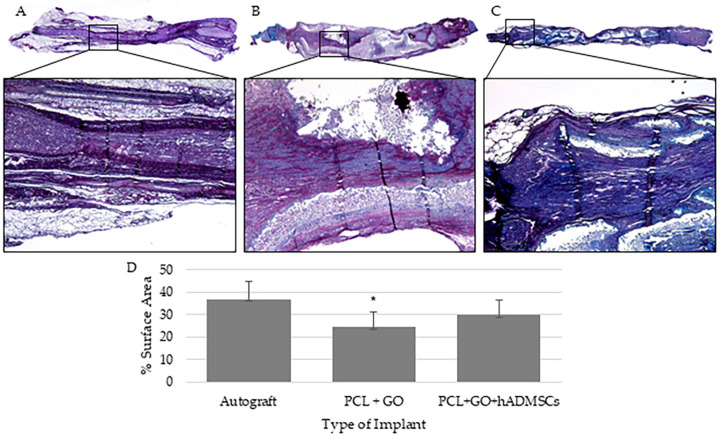
Histological analysis of sciatic nerve defect. Nerves were transected from the surgical area at 12 weeks post-surgery. Nerves were paraffin embedded, sectioned, and stained with MCOLL stain. Representative stitched images with a 5× inlay for (**A**) autograft, (**B**) PCL + 0.25% GO wrap, (**C**) PCL + GO wrap seeded with 1 million hADMSCs. (**D**) Graphical representation of the statistical analysis of percent surface area by implant type. There was no significant difference between the PCL + GO + hADMSC group as compared to the autograft, whereas there was a significant reduction in the percent surface area staining of the tissues obtained from the rats treated with the PCL + GO wrap. Left side of the stitched images shows the proximal nerve end and the right side of the stitched images shows the distal nerve end. * *p* = 0.03.

Since the autograft is considered the “gold standard”, MCOLL staining, as evaluated by percent surface area measurements, was first compared to the autograft-treated group (Figure 3). The autograft-treated rats (36.91% ± 8.00, mean ± SD) had significantly greater (*p* = 0.03) myelin surface area content as compared with that of PCL + GO-treated rats (19.05% ± 12.11). Interestingly, the autograft- and the PCL + GO + hADMSC-treated groups were similar (*p* = 0.25), suggesting that the rats treated with the combination of nerve wrap and hADMSCs showed axonal support and staining of myelin basic protein similar to that of the autograft. On the other hand, there was no difference between the PCL + GO-treated and the PCL + GO + hADMSC rats (*p* = 0.43), suggesting that the nerve wrap alone was biocompatible but not as efficacious.

### 3.4. Medial Gastrocnemius and Soleus Muscles of the PCL + GO Group Are Comparable to the Autograft Group

**Figure 4:**
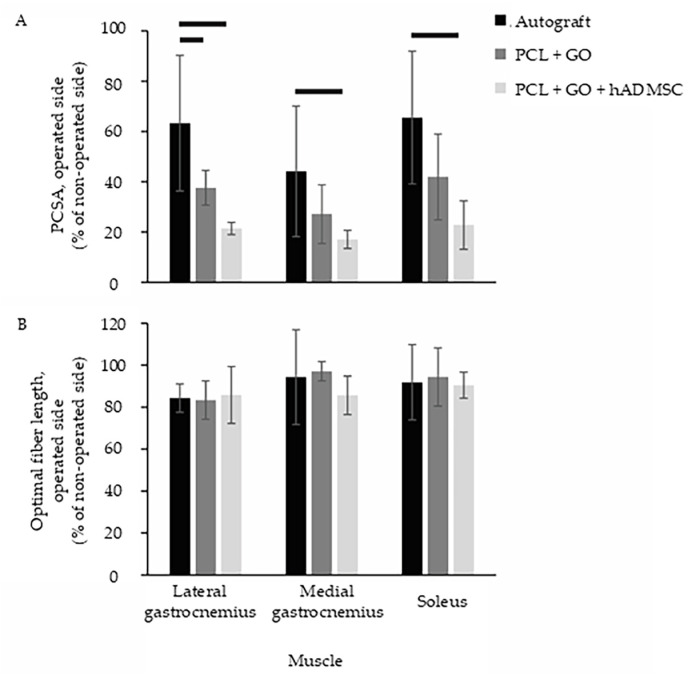
Physiologic cross-sectional area (PCSA; (**A**)) and optimal fiber length (**B**) of select muscles innervated by the sciatic nerve. All values are expressed as a percentage of values measured from the contralateral non-operated side. Error bars represent ± 1 standard deviation. Horizontal bars indicate *p* ≤ 0.05 between groups.

The physiologic cross-sectional area (PCSA) of the lateral gastrocnemius, as a percentage of the non-operated side, was significantly lower in the PCL + GO (*p* = 0.033) and PCL + GO + hADMSC (*p* = 0.001) groups compared with that of the autograft-treated group (Figure 4A). The PCSA in the PCL + GO + hADMSC group was significantly lower than that of the autograft-treated group for the medial gastrocnemius (*p* = 0.021) and soleus (*p* = 0.005). Optimal fiber length was comparable amongst all groups (Figure 4B). The PCSA is a function of both muscle mass and optimal fiber length. Given that the optimal fiber length was similar among groups, differences in the PCSA amongst the groups were driven by corresponding differences in mass. This is not surprising, since nerve injury is well known to lead to muscle atrophy (i.e., loss of muscle mass) [51,52,53].

## 4. Discussion

In this study, we report for the first time a novel polymer/nanocomposite material designed for the treatment of PNIs. We fabricated a novel PCL + GO nerve wrap to support and functionally repair damaged neural tissue and prevent neuroma formation using a rat sciatic nerve defect model. We showed that this material is cytocompatible in vitro and biocompatible in vivo with hADMSCs and could be used in combination with cells or as a safe cell delivery medical device in nerve repair. Using a combination of in vitro and in vivo assays, we report a novel PCL + GO nerve wrap which has huge potential to be used as a nerve conduit to repair PNIs. This study lays down a basic tissue engineering strategy with potential to build upon it [29].

Direct nerve repair using an autograft is one of the earliest, most traditional treatment methods for PNIs. Autografts are the current standard of treatment [5,6]. This method involves surgically repairing the damaged nerve ends by implanting a nerve segment harvested from another location in the patient’s body and suturing the respective ends together to restore the continuity of the original nerve and support the return to functionality. As a result, in this study, the group of rats treated with the autograft was used as a positive control for comparing the effects of our novel synthetic nerve graft [7,8].

Using an in vitro neural differentiation assay, we showed discrete patterns of spatial and temporal expression of S100β by hADSCs on an electrospun PCL nerve wrap coated with 0.1 mg/mL GO. S100β, a key marker of neural differentiation, is typically used to indicate trans-differentiation of naïve MSCs into Schwann cells [30]. The S100β expression in the control undifferentiated cells suggested the neural potential of naïve undifferentiated cells, whereas the increased expression in differentiated cells under cAMP induction confirmed the differentiation into Schwann-like cells, along with a discrete pattern of organization. Our data thus confirm the cytocompatibility of the biomaterials and cells, and demonstrate a potential mechanotransduction response of hADMSCs to the topographical features of the wrap [23]. Understanding the mechanism(s) of this response is very interesting, but is beyond the focus of the current study and hence will be investigated in future experiments. By translating the in vitro findings to an in vivo rat sciatic nerve defect model, we confirmed the biocompatibility of the wrap and the xenogeneic hADSCs and demonstrated that the PCL + GO + hADMSC constructs supported myelin sheath development, comparable to an autograft. The presence of a regenerated myelin sheath comparable to the autograft-treated defects identified a successful physical nerve repair in the PCL + GO + hADMSC group. Routine histological evaluation, which characterizes the tissue response to the implant, showed no signs of fibrous scar tissue or neuroma formation in either of the experimental groups, showing promise for the addition of graphene oxide to successfully prevent this complication in nerve repair [13]. The muscle analyses in the autograft-treated rats were comparable with two of the three muscle groups with the PCL + GO treatment group. Since most PNIs do not regain full function until at least 6 months post-surgery [54,55], future studies may look at tissue organization and axon repair at longer time frames (>12-weeks). Another interesting and relevant observation from these data is that using the gait and the muscle analyses, we can see some functional recovery of the nerve, although not very dramatic. This is exciting and future studies should include measuring nerve function using electrical conduction or nerve velocity types of experiments.

Even though PCL and different derivatives of graphene have been used in nanocomposites for neural tissue engineering, the combination of PCL and GO as a nerve wrap used in our study is novel [56,57,58,59]. Future experiments using controlled methods of 3D printing, varying the concentration of GO, and even using 4D printing technology to improve the surgical application, along with a more robust and efficacious response, can be implemented.

Overall, the success rates of cell-based therapies can vary widely depending on the type and composition of biomaterials and cells used, along with the method of transplantation and the timing of the intervention. Our study lays a foundation on which to build upon and improve the functional outcomes for use in tissue-engineering tools. Future experiments may work at improving both the material and cellular aspects of the nanocomposite such that these are comparable or superior to that associated with autografts.

The PCL + GO nerve wrap fabricated in this study served as a suitable delivery device for the hADMSCs. This capability can be expanded to other cell types, including Schwann cells, endothelial cells, or other stem or neural progenitor cells, if needed. Improved efficacy of cell therapy may also include priming or preconditioning cells towards a neural lineage by culturing them on the surface prior to implantation, or even culturing into more complex 3D or multi-cellular constructs. Meanwhile, identifying the timing of creating the defect and implanting the nerve wrap also may alter the treatment outcome. A 2021 review by MacKay et al. identified the significant impact that the timeframe in which the nerve repair is performed has on the long-term functional outcomes [54]. Creating the defect first and performing the surgery at a later time point may imitate a more realistic clinical scenario and outcome.

The optimization of the polymer blends and derivatives and forms of graphene, including methods of fabricating and printing scaffolds, may enhance the physical, chemical, degradative, mechanical, and conductive properties of the nerve wrap device. Changing these properties can potentially influence cell behavior and modify appropriate factors, leading to improvement in the functional outcomes [37]. For example, a 4D printed wrap may be more easily implanted around the site of a PNI caused by a crush injury. This construct could be programmed to change shape, which would minimize the manual manipulation needed by the surgeon to wrap the nerve with the material, removing the potential for additional surgical complications [38,39]. Also, increasing the concentration of GO may enhance the physicochemical properties needed for functional repair [13]. However, the exact concentration that would exhibit optimal effects before toxicity concerns overtake is unknown. There are many conflicting reports on GO cytocompatibility and cytotoxicity, with some studies reporting toxicity in filtering organs with concentrations as low as 0.4 mg/kg, while other studies reported safe outcomes in the same filtering organs with 600 mg/kg [40]. This is due to the large number of variables between studies, including surface functionalization, particle shape and size, dispersion, concentration, dosage, route of administration, and processing techniques [13,41]. Future studies may analyze toxicity in systemic immune responses and the identification of nanoparticles in filtering organs to ensure there are no adverse reactions from the parameters used within this study.

In conclusion, the novel PCL + GO nerve wrap and hADMSCs used in this study exhibited evidence of nerve repair at 12 weeks in vivo comparable to that of the clinical standard of care, the autograft. This provides a foundation on which to build upon and generate future strategies for PNI repair.

## Data Availability

All data obtained are reported in this manuscript.
